# Type 1 diabetes management in a competitive athlete: A five‐year case report

**DOI:** 10.14814/phy2.15740

**Published:** 2023-07-04

**Authors:** Roberto Cannataro, Erika Cione, Giuseppe Cerullo, Mariangela Rondanelli, Piero Micheletti, Oscar Crisafulli, Matteo Levi Micheli, Giuseppe D'Antona

**Affiliations:** ^1^ Galascreen Laboratories University of Calabria Rende Italy; ^2^ Department of Pharmacy, Health and Nutritional Sciences University of Calabria Rende Italy; ^3^ Department of Biomedical Sciences University of Padua Padua Italy; ^4^ Istituto di Ricovero e Cura a Carattere Scientifico (IRCCS) Mondino Foundation Pavia Italy; ^5^ Centro di Ricerca Interdipartimentale nelle Attività Motorie e Sportive (CRIAMS)‐Sport Medicine Centre, University of Pavia Pavia Italy; ^6^ Department of Experimental and Clinical Medicine University of Florence Florence Italy; ^7^ M. Marella Laboratory of Motor Sciences Applied to Medicine Florence Italy; ^8^ Department of Public Health, Experimental and Forensic Medicine University of Pavia Pavia Italy

**Keywords:** bioimpedance, combat sport, glycated hemoglobin, low‐carb, strength training, type I diabetes, Viet Vo Dao

## Abstract

Type I diabetes has an incidence of 15 per 100,000 people. Though it is a metabolic disorder, it can be seen in top, even professional athletes. Physical activity is recommended to manage diabetes, but there is a lack of specific knowledge on diabetes management and exercise from dedicated medical staff. This bias leads to suboptimal diabetes management, causing frequent hyper and hypoglycemia, a dysregulation of glycated hemoglobin, blood glucose out of control, and consequent needs to often intervene with extra insulin or carbohydrates. For 5 years, we followed a highly competitive male Caucasian athlete Vovinam Viet Vo Dao, with type I diabetes, aged 17. We monitored his glycated hemoglobin, the insulin drug administered, and glycemia blood level averages. We obtained, over time, a decrease in glycated hemoglobin by almost −22% and insulin administered by −37.33%, and average blood glycemia levels diminished by almost −27%. In addition, we carried out bioimpedance analysis and stratigraphy on the abdomen. Federation trainers supervised all physical training; we recorded an improvement in the general condition, underlined in particular by an increase in phase angle (from bioimpedance) of +17%.

## Graphical abstract

Over 5 years, we followed a highly competitive Vovinam Viet Vo Dao athlete with type I diabetes. We monitored his glycemic averages, glycated hemoglobin, and insulin administered (as is typical for controlling diabetes). In addition, we carried out bioimpedance analysis and stratigraphy on the abdomen. Federation trainers supervised all physical training. From a sporting perspective, the subject obtained numerous victories, two European championships, second place in the world championships, and several Italian championships. As a result of our nutritional intervention, there was a net decrease in glycated hemoglobin (−22%), insulin administered (−38%), and average blood sugar levels (−27%), with an improvement in the general condition underlined in particular by an increase in phase angle (from bioimpedance) of +17%.

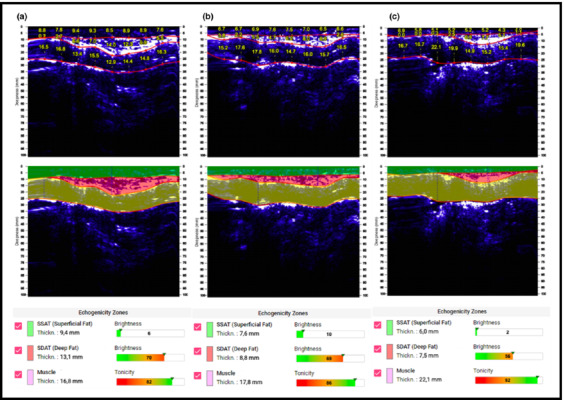

## 1 INTRODUCTION

Type I diabetes has an incidence of 15 per 100,000 people, with a global prevalence of 9.5% (Mobasseri et al., 2020). Physical activity is often recommended in the management of diabetes. The American DiabetesAssociation recommends for type I diabetes moderate‐to‐vigorous aerobic exercise, at least 150 min/week or, for adults able to run at 9.7 km/h for 25 min, 75 min/week, 3–7 days/week with no more than two consecutive days without exercise. Even high‐intensity interval training (HIIT) can be implemented, as well as resistance training (using machines, free weights, and resistance bands etc.) with moderate (1–3 sets of 15 repetitions) to vigorous (6–8 repetitions per 6–8 sets) intensity and 8–10 exercises for a minimum of two nonconsecutive days‐a weeks; it is further possible to add flexibility and balance training (Standard Medical Care in Diabetes, 2012; Colberg et al., 2016; Jaggers et al., 2016). On the contrary, these recommendations are not consistently implemented due to a fear of hypoglycemia during physical activity (Riddell et al., 2017; Yardley, 2019). The effects of regular physical activity on diabetes management are now well established. Physical activity yields a non‐insulin‐dependent hypoglycemic action resulting in a valuable tool for managing type II and I diabetes (Cockcroft et al., 2020; Turner et al., 2019; Wróbel et al., 2018). For this latter, although many professional athletes suffer from type I diabetes, there is a lack of specific literature on their experience and disease management. In a recent review of Cannata and coworkers (Cannata et al., 2020), the intake of carbohydrates (CHO) is correctly considered as nutritional therapy for athletes with both types of diabetes; however, the authors do not discuss the regulation of insulin administration for type I. In this regard, Riddel and coworkers provided guidelines on the importance of glycemic control, particularly before physical activity (Riddell et al., 2020). They recommended reducing insulin doses by up to 50% and a 1.5 g × kg body weight CHO intake, decreasing basal insulin by 20%. Avoiding hypoglycemia during or immediately after training is essential to ensure optimal performance. Medical staff dealing with patients with type I diabetes often vaguely recommend physical activity and are seldom familiar with competitive sports athletes. Since hyper or hypoglycemia may occur if glycemia levels during the training are not adequately managed. This event can happen mostly at night. Therefore, it must be emphasized how CHO intake and calorie expenditure must be considered based on the type and intensity of physical exercise. The indications given to Riddel are fundamental because some athletes can decrease their insulin dose after a pre‐workout meal or, depending on the type and intensity of training, even consider avoiding insulin administration. With this knowledge, we present the first case of professional type I athletes practicing Vovinam Viet Vo Dao (referred herein to as Vovinam).

The Vovinam is a Vietnamese martial art founded in 1938 in Hanoi; it is practiced with and without weapons. It is based on the principle relating the concepts of hard and soft. It includes training of the body as well as the mind. It uses both force and the reactions of opponents. Vovinam includes techniques involving hands, elbows, kickings, and escape‐ and levering. Both attack and defense techniques are trained, as well as combat and traditional wrestling. The techniques include punching, kicking, forms, wrestling, swords, staffs, axes, and folding fans, etc. He practiced Vovinam starting at the age of 12 years, first competing at 15.

The athlete complained of experiencing hypoglycemia during training, especially when the activity presented a significant aerobic and/or high‐intensity component, and he frequently showed nocturnal hyperglycemia. Therefore, we started following the recommendations of Riddel and coworkers (Riddell et al., 2020). The nutritional plan and the insulin administration focused on the timing and were accompanied by dietary supplements; omega‐3 fish oil, vitamin C, vitamin D, and creatine were given. This was achieved by always being in close contact with trainers. As a result, the regimen improved the subject's glycemic values, glycated hemoglobin, and insulin administration. Besides that, since Vovinam athlete competes in a sport with weight categories, body composition is of fundamental importance; for this reason, we regularly monitored through bioimpedance and stratigraphy, both the percentage and type of fat. Additionally, we used phase angle (PhA°) to determine the general fitness and hydration. Our professional type I athlete was followed for 5 years from both nutritional and training points of view, winning multiple times high competitive races.

## 2 MATERIALS AND METHODS

### 2.1 Clinical characterization

At the age of 17, we first saw a male athlete diagnosed with type I diabetes. Further, the participant's characteristics are reported: (i) weight = 67 kg; (ii) height = 166 cm; (iii) BMI = 24.3 kg/m^2^. The full dataset of the outcomes at baseline is reported in the supplementary material (TS1). This study was conducted following the Declaration of Helsinki. The protocol was approved by the Ethics Committee of the Department of Internal Medicine and Medical Therapy at the University of Pavia (Italy) with the code 001A0012. In addition, written informed consent was obtained from the participant.

### 2.2 Insulin administration

The participant used the NovoRapid FlexPen insulin aspart (Novo Nordisk Farmaceutici SPA—Rome—Italy) for rapid control of blood sugar; he started using Lantus insulin glargine (Sanofi—Paris—France), and at the age 17 (in 2016) switched to Tresiba insulin degludec (Novo Nordisk Farmaceutici SPA—Rome—Italy) for slow control; daily insulin intake, correction factor, the insulin/CHO ratio, and the glycemic mean are reported in the supplementary materials (TS2 and TS3).

### 2.3 Nutritional and supplementation plan

We reorganized the participant's nutritional plan with a scheme that provided, daily, an average of 40% of calories from CHO; the amount of CHO was lower than canonical dietetic schemes, even those used in athletes, 30% from proteins, and 30% from fats (an example is given in TS4); the adherence to the nutritional plan was monitored monthly. The 30% of professional athletes with type I diabetes use a “low‐carb” approach, meaning they have a daily CHO intake ranging from 100 to 200 g (Riddell et al., 2020). Protein intake was set according to the recommendation for competitive/professional athletes of 1.5–2.5 g/kg body weight. We strongly recommend here an intake of fiber in every meal, especially for meals richest in CHO (lunch and dinner), for a total daily amount of 20 g or more. We recommended always using low glycemic index CHO sources (whole grains, legumes, and fruit) except for meals before and after training. Together with this, we regulated insulin administration before workouts, halving the dose. The approach followed a trial and error style, similar to Calvo‐Marín et al., (2017). According to the type of training (Riddell et al., 2017 and the American College of Sports Medicine), with high intensity and duration >1 h, the administered insulin Unit (U) ranged between two and zero. The second option was chosen more often in the last 2 years; even assuming an intake of 50–60 g of CHO before workouts, we noticed that the energy demand was constant even with different workouts. The athlete typically started training with a blood sugar ranging from 120 to 160 mg/dL, and blood sugar after training was 70–90 mg/dL. In addition, we used nutritional supplements. Omega‐3 fish oil (1 g of DHA + EPA daily to achieve an anti‐inflammatory effect), vitamin C (500 mg daily, to support free radicals management and cartilage health), vitamin D (2000 IU daily only in autumn and winter, to keep levels in the normal range), creatine (5 g daily every other month) is an ergogenic supplement particularly useful in combat sport (Kreider, 2019; Kreider et al., 2017); all nutritional supplements were manufactured and supplied by 4+ Nutrition Padua‐Italy. Whey protein isolate powder was consumed when the athlete did not reach his target daily protein. Regarding nutritional supplements, in our opinion, it is essential to be careful. Mainly associated with sports and athletic practice, nutritional supplements are often used anecdotally (Cannataro et al., 2019), sometimes leading to significant side effects (Russo et al., 2016); our vision, as shown in a previous paper (Cannataro et al., 2020), is to use the minimum of nutritional supplements. A different approach was used in conjunction with competitions. The athlete needed to maintain a certain weight class, so we never applied to make weight; however, typically, 10–15 days before the competition, the workouts decreased in intensity in preparation and, therefore, the administration of insulin returned to 2 U before a workout even with a smaller quantity of CHO, (30–40 g); on the day of the competition, the management strategy was the same as for intense training (i.e., no insulin and 60 g of CHO). The athlete was monitored by a registered nutritionist every month.

### 2.4 Training program

Vovinam training includes aerobic and anaerobic exercises to develop and maintain specific technical principles of martial arts. Athletic conditioning, composed of high‐intensity interval training (HIIT) training and strength exercises, is fundamental in Vovinam training for competitors. A regular session for a competitive athlete starts with a high‐intensity specific warm‐up based on the technical movements of Vovinam, followed by strength and physical conditioning. After that, usually, the athlete performs technical training in fighting, with an extra period of muscle training at the end of the session as a cool down. As a competitive athlete, the participant trained and trained daily, alternating between purely aerobic training, mixed training with HIIT circuits (which also shows a good response in the management of type I diabetes) (Alarcón‐Gómez et al., 2021), strength training, technique training, and competition simulations, sometimes involving physical contact. A scheme of the participant's current training program is shown in Table 1.

**TABLE 1:** Weekly training program scheme. HIIT, high‐intensity interval training.

Monday	
1–2 pm	HIIT training focus arms
8–9:45 pm	Warm‐up (light HIIT)
	Fight training 30″ round 45″ rest
	Sparring
	HIIT core/push up 30″/15″
Tuesday	
1–2 PM	HIIT training focuses on core and chest
5:30–6 pm	7 km running 4.4 min/km
Wednesday	
1–2 pm	HIIT training focuses on lower body
4:30–5:30 pm	Fight training (pao and hitters) round 90″ 45″ rest
	10′ punching bag
Thursday	
1–2 PM	HIIT training focus back
8–9:45 PM	Warm‐up
	Technical session
	HIIT 30″/15″ focus core
Friday	
1–2 PM	HIIT training focus arms
8–9:45 PM	Warm‐up (light HIIT)
	Fighting simulation
	Sparring
	Tabata training
Saturday	
11–12 am	HIIT total body
	5‐6 km running 4.25 min/km or rest
Sunday	
	10 km running 4.45 min/km

### 2.5 Bioimpedance and stratigraphy analysis

Bioimpedance analysis (BIA) (Kyle, 2004; Lukaski et al., 1986) was performed with the bioimpedance analyzer, BIA 101 Anniversary (Akern Srl, Florence, Italy), using a phase‐sensitive device with alternating current at a frequency of 50 kHz. The accuracy of the BIA instrument was validated before each test session, following the manufacturer's instructions. PhA° was calculated as the arctangent of Xc/R × 180°/π. Body fat (BF) percentage was calculated using Bodygram™ software (Akern Srl). We used an ultrasound device (BX2000, BodyMetrix, IntelaMetrix, Brentwood, CA, USA) to determine the thickness of subcutaneous fat; the image's brightness represents the relative strength, or amplitude, of echoes on the computer screen. Strong reflections appear white; weaker reflections appear gray, and no echoes are black; this produces a two‐dimensional grayscale image with white borders for the skin‐subcutaneous fat and muscle‐bone interfaces and a visible but less distinct border for the fat‐muscle interface, as reported by Wagner, (2013).

## 3 RESULTS AND DISCUSSION

**FIGURE 1:**
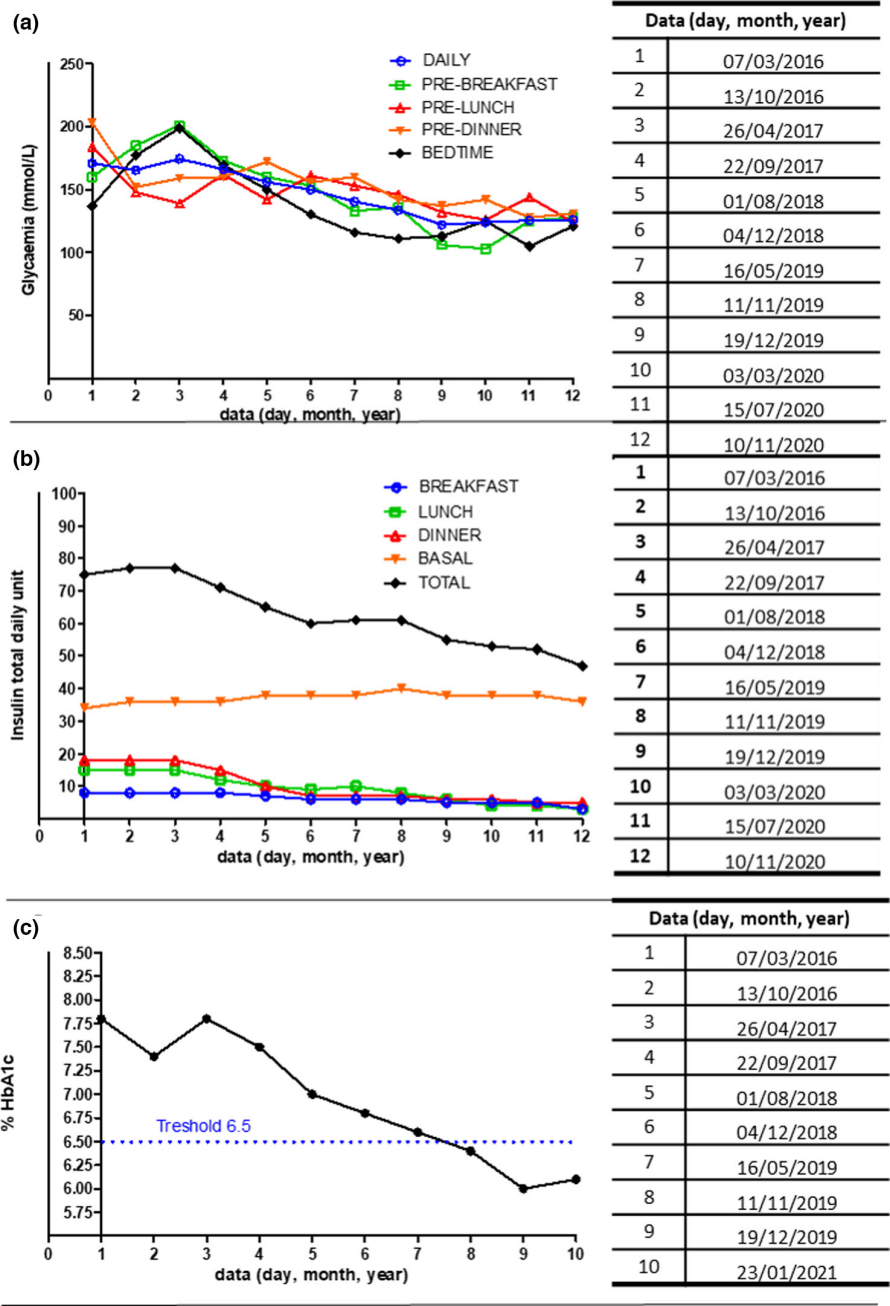
(a) Glycemia serum levels, (b) Insulin total daily unit, and (c) Glycated hemoglobin (HbA1c) percentage serum levels during the 5 years of athlete's management.

Before considering the results regarding the participant's athletic activity, we believe it is imperative to underline the results concerning the pathology at hand. In this case, after an initial period with hypoglycemia episodes during training and at night, the participant was able to reach stability in his management of type I diabetes. The glycemic averages during the study years are shown in Figure 1, panel A. It can be seen that the glycemia before main meals and overall continuously decrease, while the one going to bed and in the morning showed an initial increase and then a marked decrease, showing an average decrease of about 27%; even injected insulin in the first period showed an almost constant trend, and then decrease dosage was possible between the second and third year, reaching −37.33% at the end as it is shown in Figure 1, panel B (see also supplementary file TS2). In this sense, we underline how the basal insulin dosage was practically unchanged. However, there was a decrease of the drug in the dosage used for meals, recording a decrease of about −73% before dinner compared to the beginning (Figure 1, panel B). It should be emphasized that this was the post‐workout meal and is positively influenced by the adjustments before training. Although some studies do not agree on the efficacy of glycated hemoglobin (HbA1c) as a marker for type I diabetes, in particular with physical exercise (Kennedy et al., 2013), it remains one of the parameters used in clinical practice to monitor the normoglycemic therapy; during the study period the recorded HbA1c decrease of almost the 22%, settling on a normal average value (Figure 1, panel C). The last point to underline is that occasional nocturnal hypoglycemia was recorded; our hypothesis here is that this event was due to excess postexercise oxygen consumption (EPOC) generated by training that usually occurs in the late evening. This was managed with close monitoring of blood glucose in the evening and supplying, if needed, a supplementary carbohydrate dose of 30–40 g, which raised blood glucose to 120–130 mg/dL. We show here how the nutritional approach was fundamental in managing type I athlete performance. Attention to the type and quantity of CHO substantially made the blood sugar level more stable. Keeping glycemia in this condition is particularly important in the case of an athlete, as basal metabolism is often higher than nonathletes. Additionally, insulin regulation before training contributed to more excellent glycemic stability, confirmed by a much better insulin/CHO ratio (see supplementary TS2). On the contrary, this case report is unique, and although the results achieved were important, they should be confirmed in a larger sample. A limitation of the study is the difficulty of standardizing a procedure to re‐evaluate insulin administration and CHO intake. Therefore, the primary need in the future is to try to standardize a procedure to evaluate the variations in insulin administration in response to the quantity of CHO, even if we strongly hope that this work could represent a first step in this direction.

**FIGURE 2:**
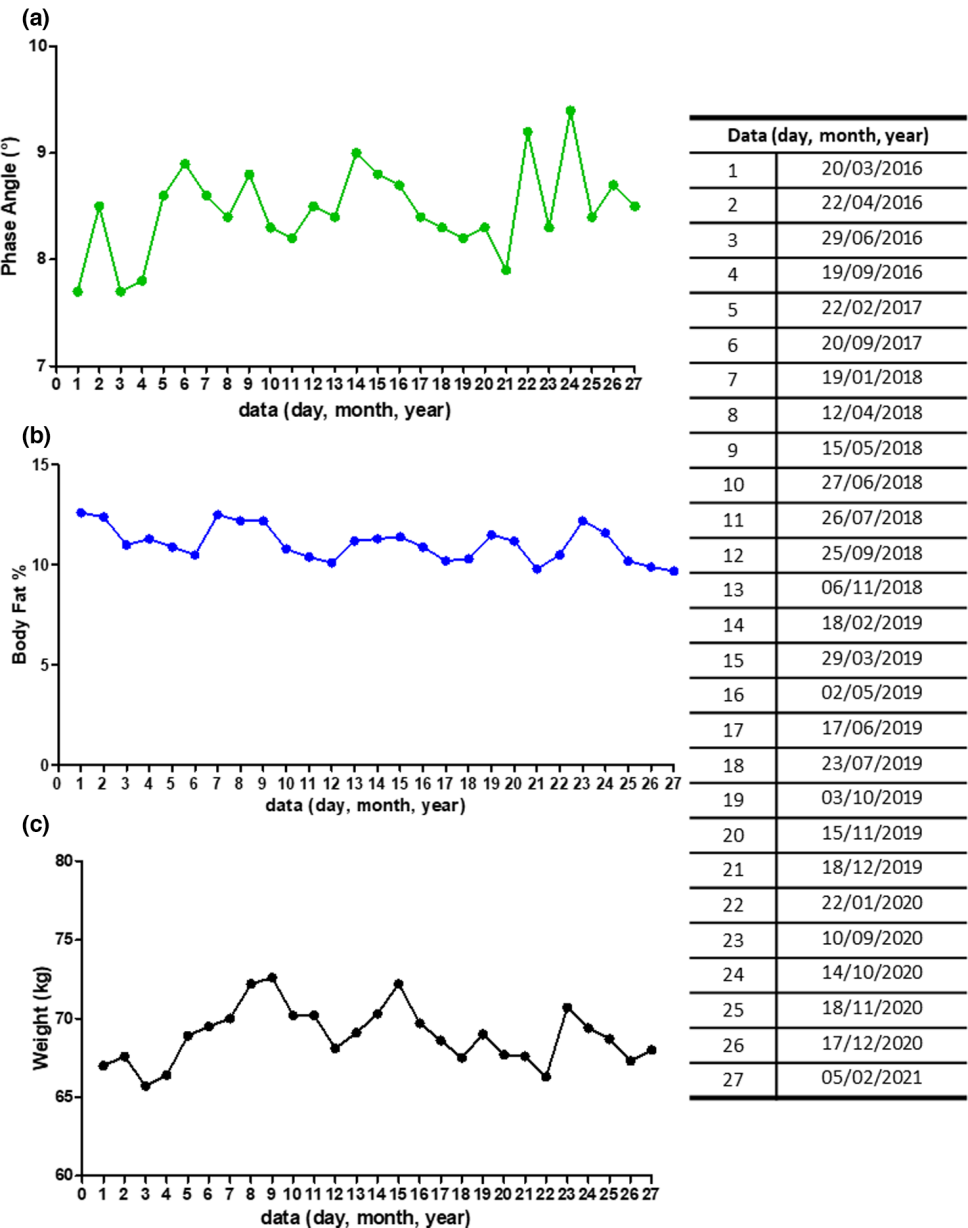
(a) Phase angle, (b) Body Fat percentage, and (c) Weight during the 5 years of athlete's management.

The goodness of BIA data, a tool widely used in the evaluation of athletes (Calella et al., 2020; Campa et al., 2022), can be seen from the phase angle (PhA°) and body fat percentage (BF%). In this concern, the BIA outstanding values were reached at the beginning of 2019, as shown in Figure 2, panel A, which displays the value of PhA°. In Figure 2, panel B is shown the BF% value (both points 14). This was in conjunction with a second place in the world championship tournament. Due to the absence of competition during the coronavirus disease 2019 pandemic, the participant asked us to apply a low‐calorie nutritional plan (see supplementary TS4). In October 2020, an excellent PhA value was recorded as a marked improvement, both in terms of general condition (Figure 2, panels A and B) and muscle mass (justifying a weight increase of 5–6 kg) (Figure 2, panel C). Another point that could be improved in future work is using dual‐energy X‐ray absorptiometry to evaluate better body composition.

**FIGURE 3:**
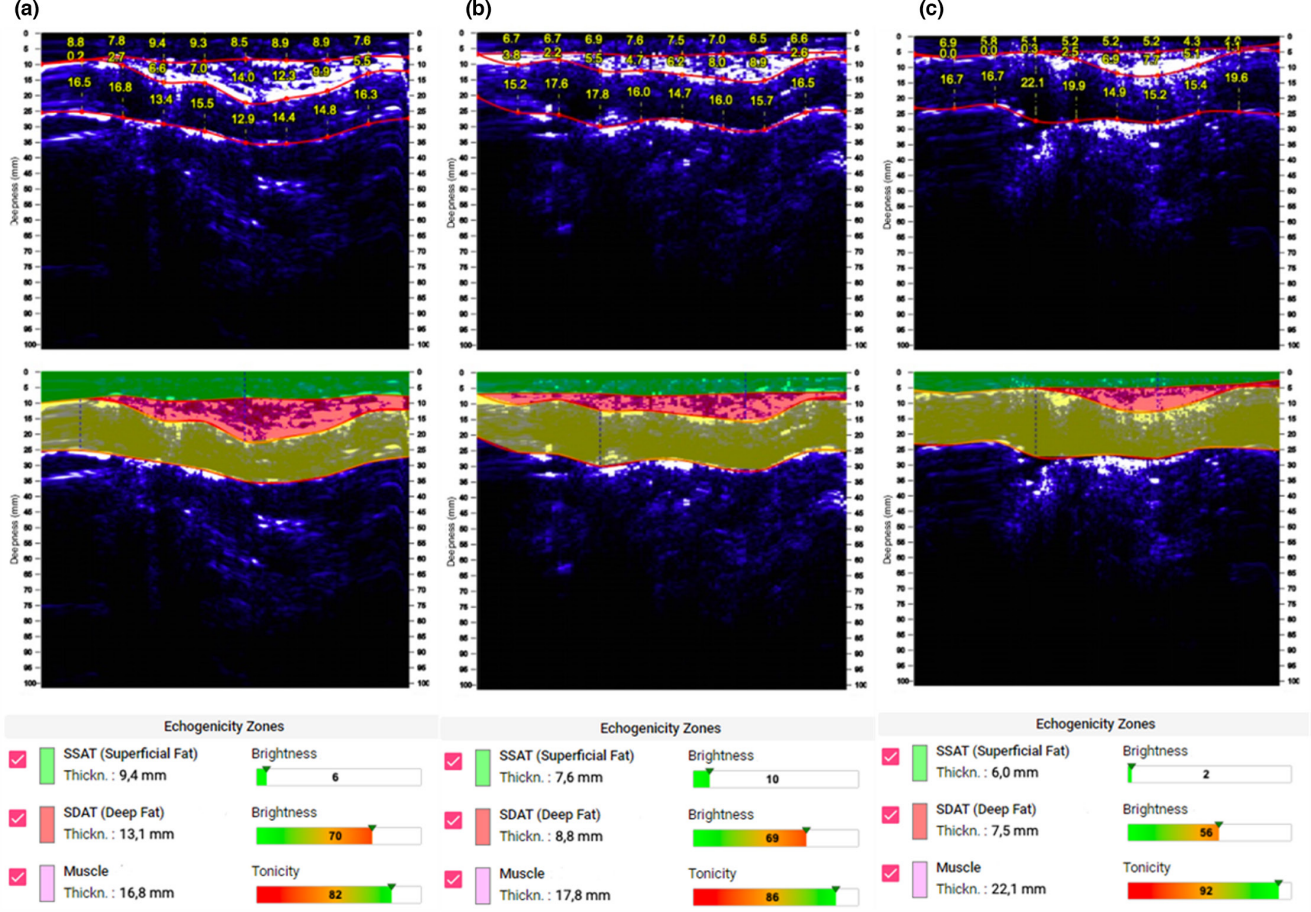
Comparative stratigraphy reports of superficial adipose tissue (SSAT) and deep (SDAT) adipose tissue of the abdomen. (a) At the begging, (b) in the middle of the nutritional plant, and (c) at the end of it. Both SSAT and SDAT decreased from 9.4 mm to 6.0 mm and 13.1 mm to 7.5 mm, respectively. Concomitantly an increase in muscle tissue from 16.8 mm to 22.1 mm was recorded.

Ultrasound stratigraphy analysis of abdomen superficial adipose tissue (SSAT) and deep adipose tissue (SDAT) measured at the begging (Figure 3, panel A), in the middle of the nutritional and training plant (Figure 3, panel B), and at the end of them (Figure 3, panel C) show thickness decrease over the time, from 9.4 mm to 7.6 mm and 6.0 mm and 13.1 mm to 8.8 mm and 7.5 mm, respectively. This was concomitantly achieved with an increase of thickness muscle tissue (MT) from 16.8 mm to 17.8 mm and 22.1 mm. These results showed a significant decrease in total subcutaneous fat of 10 mm, with an increase in muscle thickness, confirming that the weight gain was due to an increase in muscle mass. These results are positive both from a sporting point of view and, even more, regarding the long‐term diabetes management, “central” fat is related to all diabetes‐related pathologies (Ryo, 2014). In any case, the old deposition of fat is possibly due to the injection sites of insulin doses (Gibney et al., 2010). This work clearly showed that managing type I diabetes athlete's health, with careful monitoring of glycemia and appropriate adjustment made to insulin dosage/timing and diet, is possible even at a professional level. The first aspect to underline here is the improvement in all parameters relating to the management of diabetes, even in agonist practice. We must emphasize that the participant is particularly aware of his health condition so much that he is currently studying medicine. In addition, we showed that it is possible to conduct physical activity at a highly competitive level by competing on an equal footing with healthy athletes, despite the disease. Establishing a protocol to regulate insulin and CHO intake before, during, and after training would be interesting, but it would be challenging. In this sense, a collaboration between athletes, trainers, physicians, and nutritionists can be fundamental. All professional figures should be well aware of diabetes management. Plans must be adjusted to preserve health and achieve sporting excellence in close conjunction with medical staff. Devices for continuous blood glucose monitoring can help (Moser et al., 2020). In the present case, given the nature of the sport, it would be difficult and inconvenient to use it. The athlete chooses not to use a pump.

## 4 CONCLUSIONS

In conclusion, in this study, the athlete with type I diabetes was kept with a precise nutritional strategy that was able to control better glycemic fluctuation having a better performance in his competitive sport.

## AUTHOR CONTRIBUTIONS

R.C. designed and supervised the paper; M.L.M. performed and outlined bioimpedance and stratigraphy analysis; R.C. and G.C. designed and supervised the nutritional program; M.L.M., OC., and G.D.A. supervised the training program; E.C. performed statistical and biochemical analysis and interpreted the data; E.C. and G.D.A. supervised the drafting of the paper. All authors agreed to the content of the final version of the manuscript.

## FUNDING INFORMATION

No funding was obtained to produce the present work.

## CONFLICT OF INTEREST STATEMENT

The authors declare that the research was conducted without any commercial or financial relationships that could be construed as a potential conflict of interest.

## Supporting information


Supplementary Materials.
