# Supplementary material for: Type 1 diabetes management in a competitive athlete: A five‐year case report
Source: Physiol Rep. 2023 Jul 4;11(13):e15740. doi: 10.14814/phy2.15740 (PMC10319554; doi:10.14814/phy2.15740)
Supplement: Supplementary file 1 — Supplementary Materials. [file PHY2-11-e15740-s001.docx]

**Supplementary**

**TS1. Full data set of the outcomes**

| **Parameters** | **Baseline** |
| --- | --- |
| Age | 17 |
| Gender | Male |
| Weight | 67 kg |
| Height | 166 cm |
| Phase Angle (°) | 7,7 |
| Body Fat (%) | 12,6 |
| *Glycaemia average during the day (mg/dL)* | |
| Pre-breakfast | 160 |
| Pre-lunch | 184 |
| Pre-dinner | 203 |
| Bedtime | 137 |
| Average daily | 171 |
| *Insulin Therapy during the day (U)* | |
| Breakfast | 7 |
| Lunch | 15 |
| Dinner | 18 |
| Basal | 34 |
| Daily sum of Insulin | 75 |
| Correction factor (mg/dL *per* 1 U) | 24,324 |
| Insulin/CHO ratio | 4,706 |
| HbA1c (%) | 7,8 |
| *Stratigraphy data* | |
| Abdomen SSAT (mm) | 9,4 |
| Abdomen SDAT (mm) | 13,1 |
| Abdomen MT (mm) | 16,8 |

U: Unit; CHO: Carbohydrates; HbA1c: glycated hemoglobin; SSAT: Stratigraphy Superficial Adipose Tissue; SDAT: Stratigraphy Deep Adipose Tissue; MT: Muscle Tissue.

**TS2. Insulin dosage in the therapy management**

| **Day, month, year** | **Daily sum  of Insulin (U)** | **Delta % of daily sum of insulin** | **Correction factor  (mg/dL *per* 1 U)** | **Insulin/CHO ratio** |
| --- | --- | --- | --- | --- |
| 07/03/2016 | 75 | 0 | 24,324 | 4,706 |
| *Insulin switch* | | | | |
| 13/10/2016 | 75 | 0 | 24,324 | 4,706 |
| 26/04/2017 | 77 | 2,66 | 23,377 | 5,333 |
| 22/09/2017 | 77 | 2,66 | 23,377 | 5,333 |
| 01/08/2018 | 71 | -5,33 | 25,352 | 6,667 |
| 04/12/2018 | 65 | -13,33 | 27,692 | 8,000 |
| 16/05/2019 | 60 | -20 | 30,000 | 8,889 |
| 11/11/2019 | 61 | -18,66 | 29,508 | 8,000 |
| 19/12/2019 | 61 | -18,66 | 29,508 | 10,000 |
| 03/03/2020 | 55 | -26,66 | 32,727 | 13,333 |
| 15/07/2020 | 53 | -29,33 | 33,962 | 20,000 |
| 10/11/2020 | 52 | -30,66 | 34,615 | 20,000 |
| 23/01/2021 | 47 | -37,33 | 38,298 | 26,667 |

U: Unit; CHO: Carbohydrates.

**TS3. Glycaemia average during the day (mg/dL)**

| **Day, month, year** | **Pre-breakfast** | **Pre-lunch** | **Pre-dinner** | **Bedtime** | **Average daily (Ad)** | **Delta % of Ad** |
| --- | --- | --- | --- | --- | --- | --- |
| 07/03/2016 | 160 | 184 | 203 | 137 | 171 | 0 |
| *Insulin switch* | | | | | | |
| 13/10/2016 | 185 | 148 | 152 | 177 | 165,5 | -3,21 |
| 26/04/2017 | 191 | 139 | 159 | 199 | 172 | 2,04 |
| 22/09/2017 | 173 | 162 | 160 | 169,5 | 166,125 | -2,85 |
| 01/08/2018 | 160 | 142 | 172 | 150 | 156 | -8,77 |
| 04/12/2018 | 153 | 161 | 156 | 130 | 150 | -12,28 |
| 16/05/2019 | 133 | 153 | 160 | 116 | 140,5 | -17,83 |
| 11/11/2019 | 136 | 140 | 142 | 111 | 132,25 | -21,78 |
| 19/12/2019 | 112 | 132 | 137 | 113 | 123,5 | -28,65 |
| 03/03/2020 | 103 | 126 | 142 | 125 | 124 | -27,48 |
| 15/07/2020 | 132 | 144 | 128 | 105 | 127,25 | -26,60 |
| 10/11/2020 | 128 | 131 | 135 | 121 | 128,75 | -26,31 |
| 23/01/2021 | 131 | 125 | 138 | 126 | 130 | -26,60 |

| **BEFORE** |
| --- |
| **Breakfast** |
| 250-300ml of milk |
| 50-60g of breakfast cereals |
|  |
| **Snacks** |
| 60g of white bread |
| 50g of turkey breast or ham |
|  |
| **Post workout** |
| 300g of fruit (often it was not present), often it was needed to get 200-300ml of fruit juice during workout |
|  |
| **Lunch** |
| 120g of pasta or rice |
| 100g of dried beans |
| 10g of extra virgin olive oil |
| Vegetables at will |
|  |
| **Dinner** |
| 300g of potatoes |
| 200g of meat |
| 10g of extra virgin olive oil |
| Vegetables at will |
| **REVISED** |
| **Breakfast** |
| 200g of greek yogurt reduced fat (<1%) |
| 200g of seasonal fruit |
| 20g of mixed nuts (chestnut, almond, peanuts…) |
|  |
| **Snacks** |
| 200g of seasonal fruit |
| (if preworkout, add other 40-60g of whole bread according to workout type) |
| 70g of turkey breast (or 20g of isolated whey protein) |
|  |
| **Post workout** |
| 40g of dried fruit (dates, figs, apricots…) |
| 20g of isolated hydrolized whey protein |
|  |
| **Lunch** |
| 80g of whole pasta or rice |
| 100g of dried beans |
| 20g of extra virgin olive oil |
| Vegetables at will (if lunch is between two training sessions limit at 200g) |
|  |
| **Dinner** |
| 200g of potatoes |
| 200g of wild salmon |
| 20g of extra virgin olive oil |
| Vegetables at will |

**TS4. Example of nutrition plan followed by the athlete**
